# Isolation and Assessment of a Highly-Active Anti-Inflammatory Exopolysaccharide from Mycelial Fermentation of a Medicinal Fungus Cs-HK1

**DOI:** 10.3390/ijms22052450

**Published:** 2021-02-28

**Authors:** Long-Qing Li, Ang-Xin Song, Wing-Tak Wong, Jian-Yong Wu

**Affiliations:** Food Safety and Technology Research Center, Department of Applied Biology & Chemical Technology, The Hong Kong Polytechnic University, Hung Hom, Kowloon 999077, Hong Kong; longqing21.li@connect.polyu.hk (L.-Q.L.); ang-xin-3.song@polyu.edu.hk (A.-X.S.); w.t.wong@polyu.edu.hk (W.-T.W.)

**Keywords:** *Ophiocordyceps sinensis*, exopolysaccharide, fractionation, THP-1 cell culture, anti-inflammatory activity

## Abstract

The purpose of this work was to fractionate the complex exopolysaccharide (EPS) from a medicinal fungus *Ophiocordyceps sinensis* Cs-HK1 based on the molecular weight (MW) range and to assess the in vitro anti-inflammatory activity of different EPS fractions in THP-1 cell culture. The lower MW fraction (EPS-LM-1) showed a much higher anti-inflammatory activity. EPS-LM-1 was identified as a heteropolysaccharide consisting of mannose, glucose, and galactose residues with an average MW of 360 kDa. EPS-LM-1 significantly inhibited the lipopolysaccharide-induced inflammatory responses with the effective concentrations for 50% inhibition below 5 µg/mL on a few major proinflammatory markers. With such a notable in vitro anti-inflammatory activity, EPS-LM-1 is a promising candidate for the development of a new anti-inflammation therapy.

## 1. Introduction

Polysaccharides from various living organisms represent the most abundant and versatile biomaterials for a wide range of applications owing to their structural diversity, biocompatibility, and functional versatility [1]. Because of the increasing public concerns with the adverse effects of small chemical molecule drugs, natural polysaccharides have been widely explored as promising candidates for the development of novel nutraceutical products and therapeutic agents [2]. Many previous studies have shown the notable in vitro/in vivo anti-inflammatory activities of polysaccharides from various sources such as herbal plants [3], macroalga [4], and edible/medicinal fungi [5,6].

Edible and medicinal fungi or mushrooms have multiple health benefits such as anticancer, immunomodulatory, and anti-inflammatory activities, which can be attributed at least partially to the polysaccharide constituents [7]. *Ophiocordyceps sinensis* (previously named *Cordyceps sinensis*), generally known as the Chinese caterpillar fungus, is a treasured medicinal fungus in traditional Chinese medicine for health promotion and therapeutic treatment [8]. However, the wild or natural *O. sinensis* caterpillar fungus is very rare and not an affordable raw material for the extraction of polysaccharides. Instead, mycelial fermentation of *O. sinensis* fungi has become a more feasible means for the production of the fungal biomass and polysaccharides.

In previous studies, most of the *O. sinensis* fungal polysaccharides were extracted from the mycelial biomass, but very few exopolysaccharides (EPSs) from mycelial fermentation [9,10,11]. To the best of our knowledge, there is rarely any literature on the molecular characteristics and anti-inflammatory activities of EPSs produced by the *O. sinensis* fungus. Cs-HK1 is a fungal species originating from the fruiting body of a wild *O. sinensis* caterpillar fungus by Wu’s group [12]. The mycelial culture of the Cs-HK1 fungus in a liquid medium was able to produce a significant amount of EPS, which has a complex composition and wide molecular weight (MW) range [13]. In a recent study from Wu’s group, a partially purified EPS from Cs-HK1 mycelial fermentation showed significant anti-inflammatory activities in both cell culture and animal models [14]. However, the previously reported EPS was a complex mixture containing polysaccharides of different molecular weights and unknown active fractions. It is of significance to fractionate and purify the EPS and to find out the most active polysaccharide fractions, in order for better understanding of the molecular properties and anti-inflammation activity relationship as well as the mechanism of action.

The aim of the present study was to separate the complex EPS from Cs-HK1 mycelial fermentation into more uniform MW fractions and to identify, isolate, and analyze the most active anti-inflammatory EPS fraction. The in vitro anti-inflammation activity of EPS fractions was assessed in the lipopolysaccharide (LPS)-stimulated human monocytic THP-1 cell culture. The EPS in the Cs-HK1 mycelial fermentation liquid was initially separated into a higher MW and a lower MW fraction by two-step ethanol precipitation, and the lower MW fraction showing much higher in vitro anti-inflammatory activity was further fractionated and purified to homogeneity.

## 2. Results and Discussion

### 2.1. Compositions and Anti-Inflammatory Activities of Different EPS Fractions

Table 1 shows the yields, compositions, and average MW distributions of the higher MW and lower MW EPS fractions isolated from the Cs-HK1 mycelial fermentation medium by two-step ethanol precipitation. As expected, the EPS-HM attained from the first step of ethanol precipitation had a much higher average MW than the EPS-LM from the second step. EPS-LM had much higher protein content, but lower sugar content than EPS-HM. Both EPS-HM and EPS-LM were composed mainly of three monosaccharides including mannose, glucose, and galactose and EPS-HM also contained a detectable amount of ribose. The contents of the monosaccharides were very different between the two EPS fractions, with mannose being the most abundant in EPS-HM and glucose the most abundant in EPS-LM.

**Figure 1:**
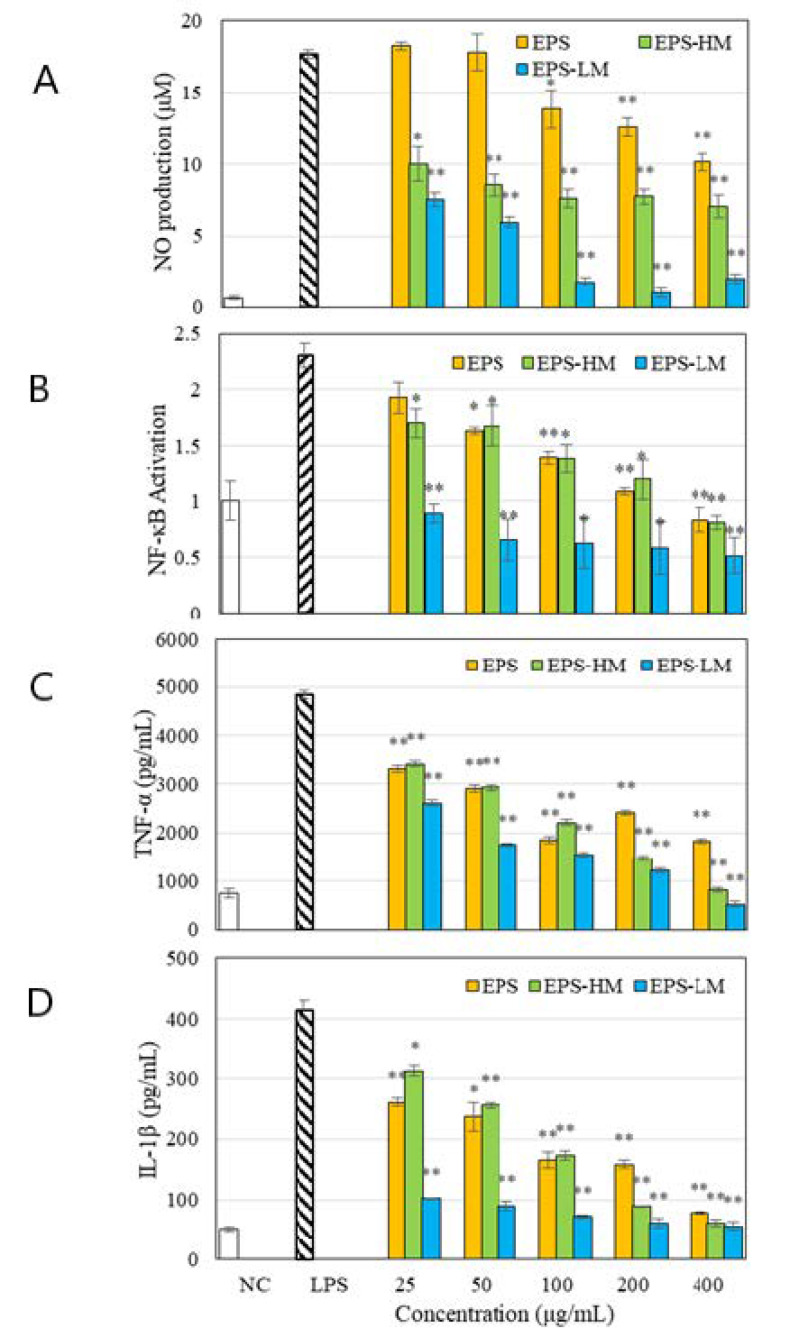
Effects of three different EPS fractions on LPS-induced inflammatory responses in THP-1 cell culture: (**A**) NO production; (**B**) NF-κB activation; (**C**) TNF-α cytokine release; (**D**) IL-β cytokine release. * and ** indicate statistically significant differences from the LPS group at *p* < 0.05 and *p* < 0.01 by the Student *t*-test, respectively.

Figure 1 shows the results of in vitro anti-inflammatory tests on EPS-HM and EPS-LM, as well as the whole EPS isolated by single-step ethanol precipitation, including NO production (Figure 1A), NF-κB activation (Figure 1B), and the release of TNF-α (Figure 1C) and IL-1β (Figure 1D) in the LPS-induced THP-1 cell culture. Of the three EPS fractions, the lower MW EPS, EPS-LM, showed the most notable inhibiting effect on all the LPS-induced inflammatory responses. As EPS-LM was the most active EPS fraction, it was chosen for further purification, characterization, and anti-inflammatory assessment.

**Figure 2:**
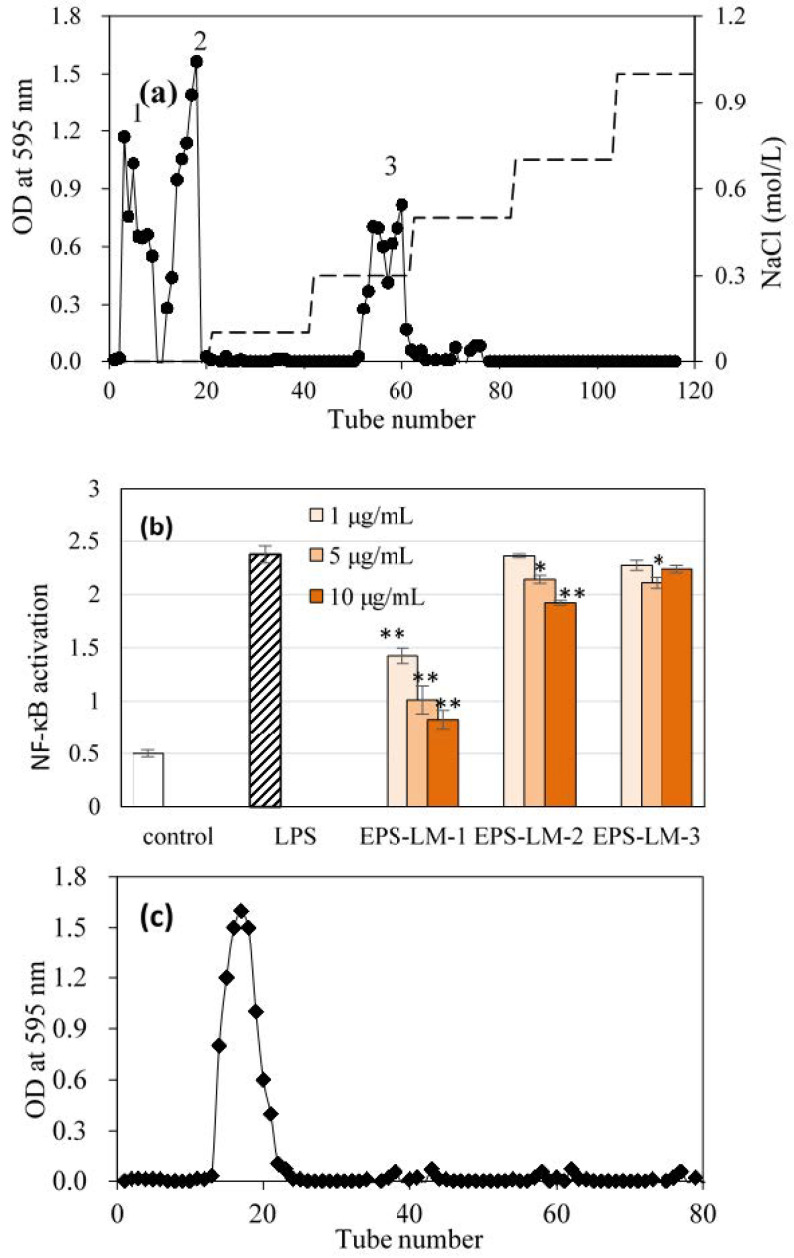
Purification and anti-inflammatory activity of EPS-LM fractions: (**a**) fractionation of EPS-LM by anion exchange on a DEAE-Sepharose column (0.1 M NaCl elution; Peaks 1, 2, and 3 representing the EPS-LM-1, -2, and -3 fractions, respectively); (**b**) anti-inflammatory activity test on EPS-LM fractions: effect on LPS-induced NF-κB activation in THP-1 cell culture (* and ** indicate statistically significant differences from the LPS group at *p* < 0.05 and *p* < 0.01 by the Student *t*-test, respectively); (**c**) GPC spectrum of EPS-LM-1 eluted from a preparative Sephadex 200 pg column.

From the anion exchange chromatograph (AEC), EPS-LM was fractionated into three major fractions, EPS-LM-1, EPS-LM-2, and EPS-LM-3 (Figure 2a). As EPS-LM-1 showed the most active in vitro anti-inflammation effect (Figure 2b), it was further purified by Superdex 200 pg (yield ~2.3% of EPS) (Figure 2c). EPS-LM-1 exhibited a single and sharp peak on HPGPC (Supplementary Materials Figure S1), corresponding to an average MW of 360 kDa by calibration. It had a total carbohydrate content of 95.4% and negligible protein content and was composed of three monosaccharide residues, Man, Glc, and Gal at a 3.9:6.9:1 mole ratio (Table 1). Compared with the partially purified EPS-LM (before AEC and GPC separations), EPS-LM-1 had a much lower content of glucose and mannose.

In a previous study by Nie et al. [15], the polysaccharide extracted from *C. sinensis* mycelium was mainly composed of glucose (95.19%) with trace amounts of mannose (0.91%) and galactose (0.61%). As reported by Sheng et al. [9], an exopolysaccharide from a cultivated *C. sinensis* was mainly composed of mannose, glucose, and galactose in a ratio of 23:1:2.6 and had an average MW of 104 kDa. A galactomannan was also isolated from natural *Cordyceps sinensis* by Wang et al. [16], which was composed of galactose (68.65%), glucose (6.65%), and mannose (24.02%). The present and previous studies have all shown that mannose, glucose, and galactose are the three major constituent monosaccharides of the polysaccharides from the *O. sinensis* fungus, while the differences in the relative contents could be attributed to the different fungal species, extraction conditions, and intracellular or extracellular location.

### 2.2. IR Spectral Characteristics of the EPS-LM-1 Structure

Table 2 summarizes the major peaks and the corresponding bonding characteristics derived from the FT-IR spectrum of the EPS-LM-1 fraction (Supplemental Data Figure S2). The large absorption peak near 3413 cm^−1^ was attributed to the -OH stretching vibration of sugar chains [17]. The weak absorption peaks at 2930 cm^−1^ and 1638 cm^−1^ were attributed to C-H stretching vibration and bound water on the sugar chains, respectively. Moreover, several weak absorption peaks around 1000 cm^−1^ indicate the vibrations of the C-O-C bond and the existence of the pyran configuration [18]. There were no absorption peaks at 1740 and 1250 cm^−1^, confirming the absence of uronic acid and sulfate groups [19]. All the spectral data showed that EPS-LM-1 contained the major functional groups in sugars. Overall, the IR spectral characteristics of EPS-LM-1 were very similar to those of the whole EPS isolated from Cs-HK1 by a single-step ethanol precipitation as reported previously [14], suggesting the similarity in the functional groups.

### 2.3. Anti-Inflammatory Activities of EPS-LM-1 in THP-1 Cell Culture

#### 2.3.1. Suppression of LPS-Induced Inflammatory Responses

**Figure 3:**
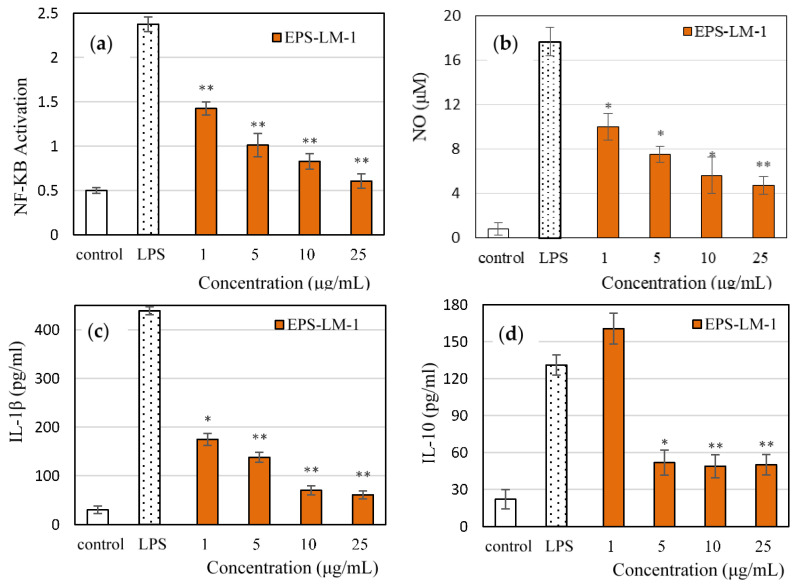
Effects of EPS-LM-1 on four inflammation responses of cells in vitro: (**a**) NO production; (**b**) NF-κB; (**c**) IL-1β; and (**d**) IL-10. Error bars for SD, n > 6. * and ** indicate statistically significant differences from the LPS group at *p* < 0.05 and *p* < 0.01 by the Student *t*-test, respectively.

As shown in Figure 3, EPS-LM-1 significantly suppressed the LPS-induced activation of NF-κB (Figure 3a), NO production (Figure 3b), and the release of IL-1β (Figure 3c) and IL-10 (Figure 3d). At the maximum concentration of 25 μg/mL, EPS-LM-1 decreased the LPS-stimulated production of NO by 76.7%, IL-1β by 92.4%, and IL-10 by 74.3%. The inhibitory effects of EPS-LM-1 on most of the inflammatory markers followed a dose-dependent trend, giving rise to the minimum effective concentrations (MECs) for 50% inhibition in Table 3. Compared with the whole EPS from a single-step ethanol precipitation reported previously [14], all three MEC values of EPS-LM-1 were notably lower, implying that EPS-LM-1 was much more potent in the inflammatory responses.

The NF-κB signaling pathways can be activated by LPS in the granulocytes, monocytes, macrophages, and natural killer (NK) cells via upregulation of the DNA-binding activity of NF-κB [20,21]. NO, IL-1β, and IL-10 are important signaling elements for mediating the host immune and inflammatory responses, especially during infection and trauma [2,22]. Therefore, the experimental results suggest that EPS-LM-1 inhibited the activation of the inflammatory NF-κB pathway and the secretion of inflammatory cytokines.

#### 2.3.2. Suppression of Inflammatory Protein Expression by EPS-LM-1

**Figure 4:**
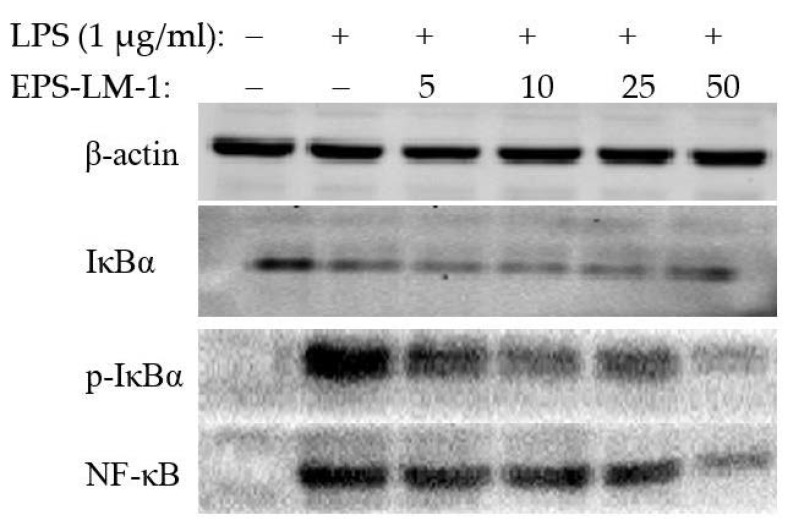
Effects of EPS-LM-1 on the activation of the NF-κB signaling pathway in LPS-stimulated THP-1 cells (β-actin: internal reference).

Figure 4 shows the LPS-stimulated expression levels of inflammatory proteins in the NF-κB pathway, including NF-κB, IκBα, and phospho-IκBα (NF-kappa-B inhibitor alpha) in THP-1 cells and the effects of EPS-LM-1 treatment. EPS-LM-1 significantly inhibited the phosphorylation and proteolytic degradation of IκBα. Although the expression of IκBα was not notably changed, the expression of p-IκBα was significantly downregulated at sufficient EPS-LM-1 concentrations. Concomitantly, LPS-induced NF-κB activation was strongly inhibited with EPS-LM-1, suggesting that EPS-LM-1 suppressed the LPS-induced inflammatory responses of THP-1 cells by inhibiting the NF-κB pathway.

**Figure 5:**
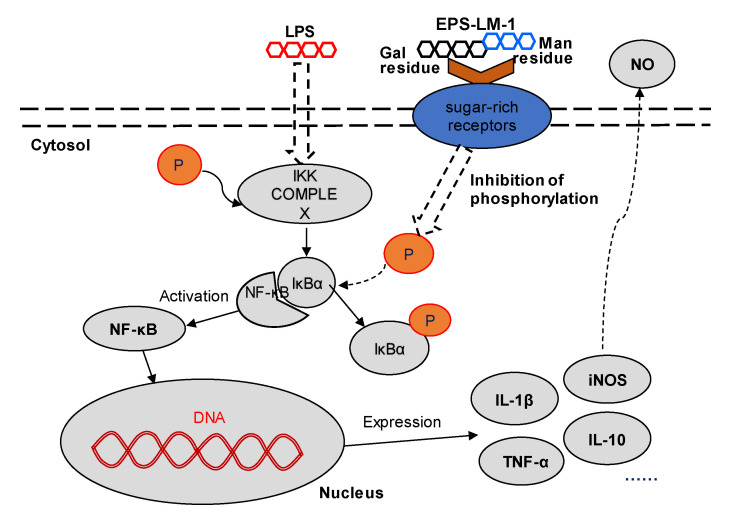
Proposed signaling pathway for the inhibition of LPS-induced inflammatory responses by EPS-LM-1 in THP-1 cells.

The results from many previous studies in cell culture and animal models have suggested that homo- and hetero-polysaccharides modulate the cell immunity and inflammatory responses through different membrane receptors, particularly the sugar-rich or glycan binding receptors such as the mannose receptor (MR) to recognize the mannose at the polysaccharide terminals [23] and a specific galactose lectin on the surface of inflammatory macrophages involved in the interaction of relative inflammatory glycoprotein regulation [24,25]. NF-κB is widely distributed in animal tissues, participating in the transcriptional regulation of many inflammatory genes in response to stimulation, and also plays a key role in a variety of physiological and pathological processes such as host immunity, cell proliferation, and apoptosis [26,27]. Generally, the inactive form of the NF-κB complex associated with IκBα is silenced in the cytoplasm, which prevents the translocation of NF-κB to the nucleus. However, LPS treatment of the cells triggers the IKK-mediated phosphorylation and degradation of IκBα, leading to the release of the NF-κB complex and its translocation to the nucleus, which ultimately results in the transcription of inflammation-related genes [28]. The results revealed that the upregulated expression of p-IκBα in LPS-stimulated THP-1 cells was significantly inhibited by treatment with EPS-LM-1 in a dose-dependent manner. In addition, the LPS-induced secretion of IL-1β, IL-10, and NO was also markedly reversed by EPS-LM-1. Therefore, it is proposed that EPS-LM-1 inhibited the LPS-stimulated inflammatory responses in the THP-1 cells via suppression of the NF-κB pathway, as illustrated in Figure 5.

#### 2.3.3. Comparison of the Anti-Inflammatory Activity of EPS-LM-1 with Other Polysaccharides

The three EPS fractions isolated from the Cs-HK1 mycelial fermentation medium, EPS, EPS-LM, and EPS-HM showed different levels of inhibition on the LPS-stimulated inflammatory responses (Figure 1). The different activity levels among these three EPSs may be attributed to their differences in chemical composition (total carbohydrate and protein content, monosaccharide composition), MW range, and structural characteristics. In comparison with EPS-HM, EPS-LM had a much lower MW and was perhaps more accessible to the cells, producing a faster and higher activity. The functions and bioactivities of a polysaccharide are dependent on a number of structural characteristics such as monosaccharide composition, linkage pattern, degree of branching, and chain length or molecular weight [5,6,11]. In addition, these basic molecular characteristics of a polysaccharide are chief factors influencing the hydrocolloidal properties of polysaccharide aggregates in an aqueous solution that are formed through complex interactions among the biomacromolecules and the polymer chains. The hydrocolloidal properties determine the solution properties such as viscosity and solubility, which can also affect the biological functions significantly.

Compared with many polysaccharides from various sources that have been shown being active in suppressing the LPS-stimulated inflammatory responses in the literature, EPS-LM-1 is among the most potent. For example, a crude polysaccharide from a cultured *C. sinensis* fungus inhibited LPS-stimulated IL-1β effectively in human proximal tubular epithelial cells (HK2 cells) at 750 μg/mL [6]. Lentinan, a well-known fungal β-glucan claimed to have notable anticancer and immunomodulation activity, inhibited LPS-induced IL-8 expression in Caco-2 cells significantly at 500 μg/mL [29]. The effective concentration ranges were all much higher than the effective dosage of EPS-LM-1 in the present study (MEC for 50% inhibition < 5 μg/mL). This suggests that EPS-LM-1 is a significantly more potent and active anti-inflammatory than other crude or purified polysaccharides from edible and medicinal fungi reported previously.

## 3. Materials and Methods

### 3.1. Cs-HK1 Mycelial Fermentation and Crude EPS Isolation

The Cs-HK1 fungus was originally isolated from the fruiting body of a wild *C. sinensis* caterpillar fungus by Wu’s group and preserved in mycelial culture on a solid substrate as reported previously [12]. For EPS production, Cs-HK1 mycelial fermentation was carried out in shake flasks in a liquid medium consisting of 40 g/L glucose, 5 g/L peptone, 1 g/L KH_2_PO_4_, 0.5 g/L MgSO_4_·7H_2_O, and 10 g/L yeast extract on a shaking incubator at 200 rpm and 20 °C for 7 days. The fermentation liquid was then centrifuged (12,000 rpm; 20 min; 4 °C) to attain a solid-free medium, and the supernatant was collected for EPS isolation by ethanol precipitation. More details of the mycelial fermentation and ethanol precipitation of EPS have been reported previously [12,14]. In the present study, a two-step ethanol precipitation was performed using 40% (*v*/*v*) ethanol in the first step and 80% (*v*/*v*) ethanol in the second step. In the first step, ethanol was added slowly to the supernatant medium to a final concentration of 40% (*v*/*v*) (=2:3 volume ratio) with stirring and then kept in stationary condition at 4 °C for 12 h. The mixture was centrifuged at 10,000 rpm for 20 min to separate the solid from the liquid. In the second step, the ethanol was added to the liquid supernatant at a 10:3 volume ratio to make up a total volume ratio of 4:1 (2:3 in the 1st step + 10:3 in the 2nd step) and the final ethanol concentration of 80% (*v*/*v*). All precipitates collected after the centrifugation were re-dissolved in water and lyophilized, yielding the crude EPS fraction in the higher MW range (EPS-HM) from the first step and the lower MW range (EPS-LM) from the second step, respectively.

### 3.2. Purification of EPS-LM

**Figure 6:**
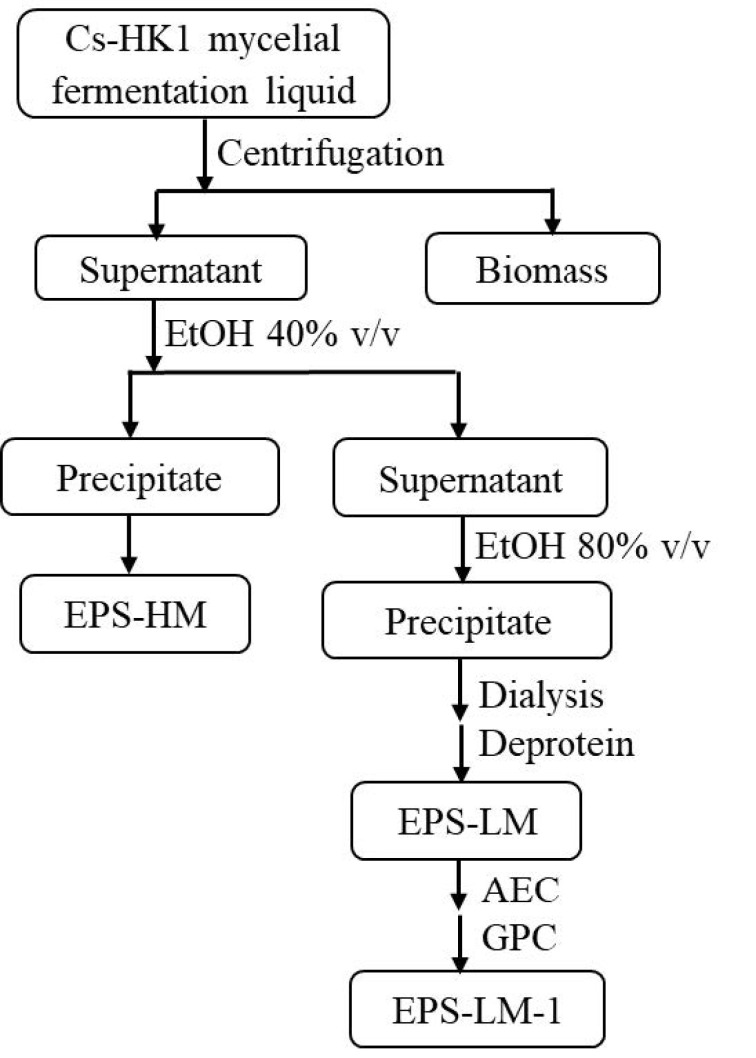
Experimental procedure for the isolation of EPS from Cs-HK1 mycelial fermentation by two-step ethanol (EtOH) precipitation and further fractionation and purification to attain EPS-LM-1 (AEC: anion exchange chromatograph; GPC: gel permeation chromatograph).

The lower MW fraction EPS-LM attained from the above two-step ethanol precipitation was treated repeatedly with Sevag reagent to remove the protein content [30]. The solution was then dialyzed against a 3500 Da MW cut-off membrane for 48 h, concentrated by evaporation, and then lyophilized. About 500 mg of the partially purified EPS-LM was re-dissolved in 2 mL distilled water (250 mg/mL) and fractionated by anion exchange chromatography (AEC) on a DEAE-Sephadex A-25 column (2.6 × 100 mm) (Pharmacia) and eluted stepwise with distilled water and 0.1 M NaCl at a flow rate 0.5 mL/min. Polysaccharide in the elute was detected by the anthrone agent (Sigma). A major fraction was collected during water elution from three fractions and further purified by gel permeation chromatograph (GPC) on a Superdex 200 pg column (2.6 × 60 cm, Cl^−^; Whatman) eluted with 0.3 M NH_4_HCO_3_ at a flow rate of 0.3 mL/min. The eluate was collected for detection of polysaccharide by the anthrone method. The main polysaccharide fractions were pooled, concentrated, dialyzed, and lyophilized, yielding the final purified fraction EPS-LM-1. Figure 6 shows the overall procedure for EPS isolation by two-step ethanol precipitation and further fractionation and purification of EPS-LM through AEC and GPC.

### 3.3. Analysis and Characterization of EPS-LM-1

#### 3.3.1. Analysis of the Molecular Weight

The MW distribution was analyzed by high-performance gel permeation chromatography (HPGPC) as reported previously [14]. A Waters HPGPC instrument was used consisting of a Waters 1515 isocratic HPLC pump, a Waters 2414 refractive index (RI) detector, and a Waters 2998 UV detector. Two columns were used in series, the Ultrahydrogel TM 2000 and Ultrahydrogel TM 500 column (Waters). Water was used as the mobile phase. The MW calibration was performed with several dextran MW standards ranging from 1.0 to 670 kDa (Sigma-Aldrich, St. Louis, MO, USA). The sample and standards were dissolved in DI water and filtered through a 0.22 μm membrane before being injected into the instrument system.

#### 3.3.2. Analysis of the Chemical Composition

The total carbohydrate content was determined by the anthrone test using glucose as a standard and the total protein by the Lowry method using bovine serum albumin (BSA) as a standard [8,12]. The monosaccharide composition was analyzed by the 1-phenyl-3-methyl-5-pyrazolone (PMP)-HPLC method as reported previously [14]. In brief, the polysaccharide samples (1–2 mg) were completely hydrolyzed with 2 M trifluoroacetic acid (TFA) at 110 °C for 4 h. The hydrolysate was dried by evaporation under vacuum and then derivatized in 450 μL PMP solution (0.5 M in methanol) and 450 μL of 0.3 M NaOH at 70 °C for 30 min. The reaction was stopped by neutralization with 450 μL of 0.3 M HCl, followed by extraction with chloroform (1 mL, 3 times). The extract solution was applied to HPLC analysis with an Agilent ZORBAX Eclipse XDB-C18 column (5 μm, 4.6 × 150 mm) on an Agilent 1100 instrument with a G1312A Bin pump and a UV detector.

#### 3.3.3. FT-IR Analysis

IR spectroscopy was performed at room temperature in the range of 4000–500 cm^−1^ at 4 cm^−1^ resolution on an Avatar 360 Fourier-transform infrared spectroscopy (FT-IR) (Thermo Nicolet, Cambridge, UK). Briefly, the dry solid sample (2 mg) was ground with KBr powder, then pressed into a pellet and measured on the FT-IR spectrometer.

### 3.4. In Vitro Anti-Inflammation Activity Assay

#### 3.4.1. THP-1 Cell Culture

The THP-1-dual cell line was purchased from InvivoGen (USA), which was derived from the human THP-1 monocyte cell line by stable integration of two inducible reporter constructs (NF-κB-secreted embryonic alkaline phosphatase (SEAP) and IRF-Lucia reporter monocytes). It resembles the human monocyte in many aspects such as morphology, secretory products, expression of membrane antigens, and even expression of genes involved in lipid metabolism. Compared with the native human monocytes, THP-1 has an additional advantage of a homogeneous population, which greatly facilitates in vitro studies [31]. The THP-1 cell culture was maintained in RPMI 1640 supplemented with 10% fetal bovine serum (FBS) and penicillin (100 U/mL) and streptomycin (100 μg/mL) with extra 25 mM HEPES (Sigma-Aldrich) and 100 μg/mL Normocin (InvivoGen, San Diego, CA, USA), at 37 °C in a humidified incubator with 5% CO_2_. Zeocin™ and blasticidin (InvivoGen, San Diego, CA, USA) were added to the growth medium every other passage to maintain the selection pressure, and the passage number was kept below 20.

#### 3.4.2. Analysis of NF-κB Activation and Nitric Oxide Production after LPS and EPS Treatment

NF-κB activation was determined by assessing the NF-κB-induced secreted embryonic alkaline phosphatase (SEAP) activity of THP-1 cells using Quanti-Blue agent (InvivoGen) according to the InvivoGen protocol, as reported previously [14]. In brief, the THP-1 cells were firstly incubated in 96 well flat-bottom tissue culture plates (~2 × 10^5^ cells/well) for 48 h and then treated with 200 ng/mL lipopolysaccharide (LPS) (Sigma-Aldrich) or 200 ng/mL LPS + 25–400 µg/mL of EPS fractions. A control group was included without LPS and EPS treatment. After 48 h of incubation, the SEAP activity in the culture medium was determined with Quanti-Blue by measuring the absorbance at 625 nm with a spectrophotometer. The NF-κB activation was represented by the absorbance.

Nitric oxide (NO) concentration in the culture medium was determined by a nitrite detection kit (Beyotime, Catalog #S0021, Shanghai, China). All the samples were tested at least in triplicate according to the manufacturer’s instructions.

#### 3.4.3. Measurement of Cytokines by ELISA

THP-1 cells (5 × 10^5^ cells/well) were seeded in 24 well plates and incubated for 24 hm then treated with LPS to induce the secretion of inflammatory related cytokines (TNF-α, IL-1β, and IL-10) or treated with LPS+EPS for 48 h. After incubation, the concentrations of TNF-α, IL-1β, and IL-10 in the culture medium were determined using DuoSet ELISA kits (TNF-α kits, Catalog #KE0068, Proteintech, Rosemont, IL, USA; IL-1β kits, Catalog #RAB0273, Sigma-Aldrich, Steinheim, Germany; IL-10 kits, Catalog #DY217B-05, R&D Systems, Minneapolis, MI, USA) according to the manufacturer’s instructions.

#### 3.4.4. Western Blot Analysis

After treatment with LPS and EPS-LM-1, the THP-1 cells were harvested and washed with cold phosphate-buffered saline (pH 7.4). The total cytoplasmic protein (for the detection of NF- κB, IκBα) was extracted from the cells using the ice-cold cell lysis reagent (Catalog # R0278, Sigma-Aldrich, Germany), and β-actin was included as an internal reference. The protein content in the supernatant was determined using the BCA protein assay kit (Catalog #23225, Thermo, Rockford, IL, USA). An equivalent amount of lysate protein (20 μg per lane) was separated by 10% SDS-PAGE and transferred to a polyvinylidene difluoride membrane. The membrane was blotted with 5% (*w*/*v*) skimmed milk, and the blots were incubated with specific primary antibodies overnight at 4 °C, followed by incubation with horseradish peroxidase-conjugated secondary antibody. Finally, the blots were probed using enhanced chemiluminescence and autoradiographed.

### 3.5. Statistical Analysis

All data were expressed as the mean ± standard deviation (SD) from at least triplicate experiments (*n* ≥ 3). Statistical analysis was performed using SPSS v.16.0 for Windows (SPSS Inc., Chicago, IL, USA). The independent-samples Student test was performed for comparison of the differences among the treatment groups.

## 4. Conclusions

In the present study, a highly active polysaccharide EPS-LM-1 with a homogenous MW of 360 kDa was isolated and purified from the crude exopolysaccharide (EPS) produced by Cs-HK1 mycelial fermentation. EPS-LM-1 was a heteropolysaccharide composed of glucose, mannose, and galactose residues in the main chain and a galactose residue in the side chain. It showed remarkable activity against the LPS-induced activation of the NF-κB pathway and the related inflammatory responses in THP-1 cells. The inhibiting effects were very potent with minimum effective concentrations as low as 2–5 µg/mL on some major pro-inflammatory factors including NF-κB, NO, and IL-1β. The modulation of the NF-κB signaling pathways by inhibiting the phosphorylation of IκBα was detected as a possible route for these in vitro anti-inflammatory effects of EPS-LM-1. The results from the present study are encouraging for further research and development of the Cs-HK1 EPS as new therapeutics for the treatment of inflammation-related diseases. It is worthwhile to further characterize the molecular structure of EPS-LM-1 and to investigate the structure-activity relationship, as well as the anti-inflammatory effects of action in animal models.

## Figures and Tables

**Table 1:** Yield and composition of exopolysaccharide (EPS) fractions isolated from the Cs-HK1 fermentation medium by two-step ethanol precipitation and further fraction of EPS-lower MW (LM). HM, higher MW. ^a^. Original GPC results shown in Supplemental Data Figure S1.

**Fraction**	**Yield (g/L)**	**Sugar (wt %)**	**Protein (wt %)**	**MW in kDa** **(GPC Peak Area%) ^a^**
EPS-HM	2.27 ± 0.19	64.5 ± 5.70	9.0 ± 0.39	6250 (84.76%) 18.5 (15.24%)
EPS-LM	0.93 ± 0.15	25.5 ± 0.60	20.1 ± 0.06	360 (68.7%) 39.2 (12.3%) 1.61 (19.0%)
EPS-LM-1	0.074 ± 0.003	100	-	360 (100%)
Monosaccharide composition (molar ratio)				
**Fraction**	**Mannose**	**Ribose**	**Glucose**	**Galactose**
EPS-HM	1.71	0.09	0.94	1
EPS-LM	7.76	-	13.87	1
EPS-LM-1	3.88	-	6.93	1

**Table 2:** FT-IR peaks and bonding characteristics of EPS-LM-1.

Peak Wavenumber (cm^−1^)	Functional Groups
3400	Axial stretch of -OH group
2930	Weak C-H stretching vibration
1638, 1539	Asymmetric and symmetric vibration of the ring stretching of the carboxylate group, respectively.
1380	OH bending vibration
1065	Pyranoside (e.g., in the glucose residues)

**Table 3:** Relative potency indexes of EPS-LM-1 compared with EPS [14] on three major inflammatory markers (MEC: minimum effective concentration for 50% inhibition).

Pro-Inflammation Factors	MEC (μg/mL)	
	EPS	EPS-LM-1
NF-κB	67.6	4.77
NO	123.3	3.74
IL-1β	183.6	1.90

## Data Availability

Not applicable.

## Supplementary Materials

Supplementary materials can be found at https://www.mdpi.com/1422-0067/22/5/2450/s1.
