# Supplementary material for: Isolation and Assessment of a Highly-Active Anti-Inflammatory Exopolysaccharide from Mycelial Fermentation of a Medicinal Fungus Cs-HK1
Source: Int J Mol Sci. 2021 Feb 28;22(5):2450. doi: 10.3390/ijms22052450 (PMC7957654; doi:10.3390/ijms22052450)
Supplement: Supplementary file 1 [file ijms-22-02450-s001.pdf]

Supplemental data

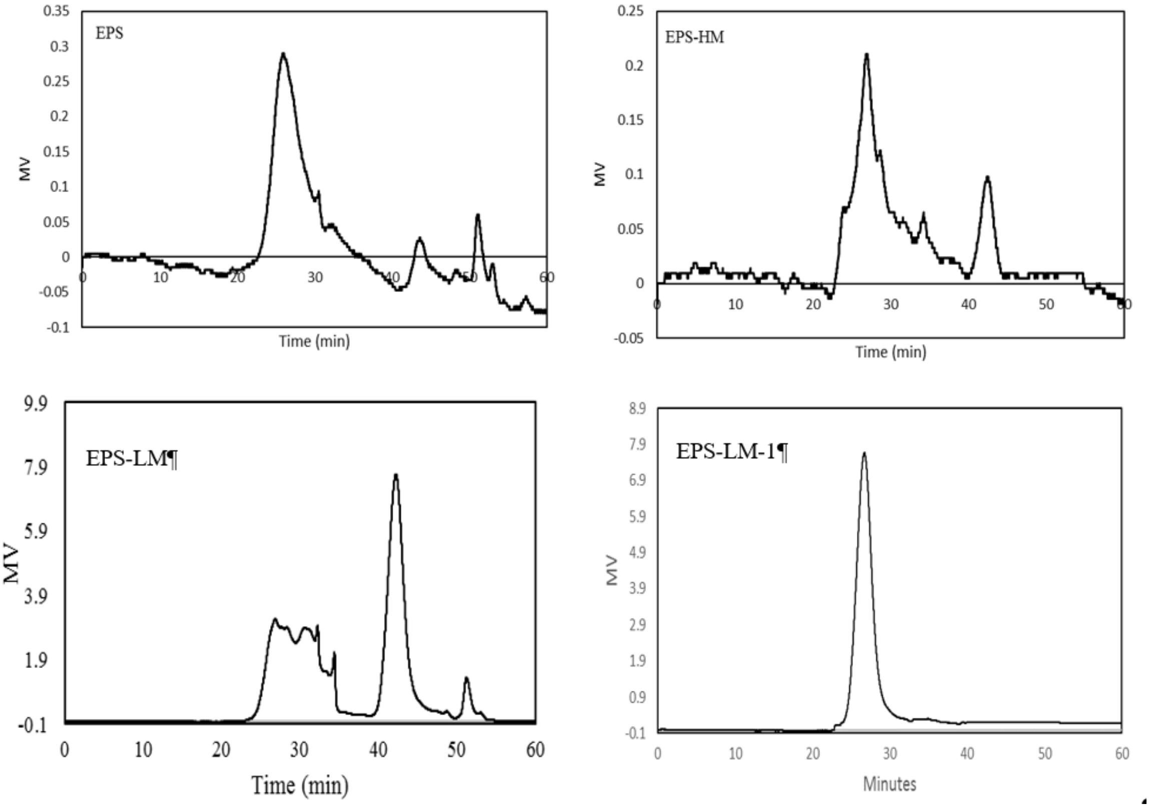

Figure S1. HPGPC profiles of different fractions.

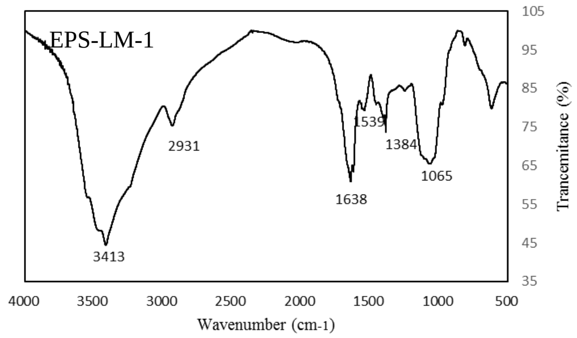

Figure S2. FT-IR spectrum of EPS-LM-1.
